# Highly sensitive sandwich immunoassay and immunochromatographic test for the detection of *Clostridial* epsilon toxin in complex matrices

**DOI:** 10.1371/journal.pone.0181013

**Published:** 2017-07-11

**Authors:** Cécile Féraudet-Tarisse, Christelle Mazuet, Serge Pauillac, Maren Krüger, Caroline Lacroux, Michel R. Popoff, Brigitte G. Dorner, Olivier Andréoletti, Marc Plaisance, Hervé Volland, Stéphanie Simon

**Affiliations:** 1 Service de Pharmacologie et Immunoanalyse (SPI), CEA, INRA, Université Paris-Saclay, Gif-sur-Yvette, France; 2 Unité des Bactéries anaérobies et Toxines, Institut Pasteur, Paris, France; 3 Biological Toxins, Centre for Biological Threats and Special Pathogens, Robert Koch Institute, Berlin, Germany; 4 INRA, UMR 1225, Interactions Hôtes Agents Pathogènes, Ecole Nationale Vétérinaire de Toulouse, Toulouse, France; New York State Department of Health, UNITED STATES

## Abstract

Epsilon toxin is one of the four major toxins of *Clostridium perfringens*. It is the third most potent clostridial toxin after botulinum and tetanus toxins and is thus considered as a potential biological weapon classified as category B by the Centers for Disease Control and Prevention (CDC). In the case of a bioterrorist attack, there will be a need for a rapid, sensitive and specific detection method to monitor food and water contamination by this toxin, and for a simple human diagnostic test. We have produced and characterized five monoclonal antibodies against common epitopes of epsilon toxin and prototoxin. Three of them neutralize the cytotoxic effects of epsilon toxin *in vitro*. With these antibodies, we have developed highly sensitive tests, overnight and 4-h sandwich enzyme immunoassays and an immunochromatographic test performed in 20 min, reaching detection limits of at least 5 pg/mL (0.15 pM), 30 pg/mL (0.9 pM) and 100 pg/mL (3.5 pM) in buffer, respectively. These tests were also evaluated for detection of epsilon toxin in different matrices: milk and tap water for biological threat detection, serum, stool and intestinal content for human or veterinary diagnostic purposes. Detection limits in these complex matrices were at least 5-fold better than those described in the literature (around 1 to 5 ng/mL), reaching 10 to 300 pg/mL using the enzyme immunoassay and 100 to 2000 pg/mL using the immunochromatographic test.

## Introduction

Epsilon toxin (ε toxin) is one of the four major toxins produced by *Clostridium perfringens* together with alpha, beta and iota. Depending on their ability to produce one or more of these four lethal toxins, *C*. *perfringens* strains are classified into five toxinotypes (A, B, C, D and E), ε toxin being synthesized by *C*. *perfringens* types B and D [1]. This is a Gram-positive, anaerobic, sporulating bacterium which also produces many other toxins (up to 17) not used for classification, making it the largest producer of toxins of any bacteria [2].

Except for the chromosomal alpha-toxin encoding gene, all *C*. *perfringens* toxins used for toxinotyping, and in particular ε toxin, are encoded by genes located on large plasmids [3–5]. This accounts for the large genetic diversity in *C*. *perfringens* strains because of the mobility of plasmids, which can be acquired, rearranged or lost. Thus, a *C*. *perfringens* strain can change from one toxinotype to another by acquisition or loss of a toxinogenic plasmid.

Epsilon toxin is secreted as a poorly active single-chain protein, known as prototoxin (296 amino acids, 33 kDa). The prototoxin is converted by proteolytic enzymes (produced by *C*. *perfringens* and/or present in its environment) to the > 1,000-fold more toxic form, by cleavage of the 10–13 N-terminal amino acids and 22–29 C-terminal residues depending on the protease [3,6]. These cleavages result in a significant reduction in molecular weight (from 33 kDa to approximately 28.6 kDa) and a substantial decrease in the pI value (from 8.02 to 5.36), which is probably accompanied by conformational changes. C-terminal processing was also shown to be essential for heptamerization of ε toxin, a common feature of pore-forming units [7].

*C*. *perfringens* types B and D, which produce ε toxin, cause dysentery, enteritis and enterotoxemia, mainly in sheep and goats, and these diseases are of significant economic importance. The natural sources of *C*. *perfringens* are anaerobic habitats with organic nutrients, particularly soils, aquatic sediments, litters or cadavers. This bacterium may also be resident in the digestive tract where it can be found in low numbers (< 10^3^ CFU/g) in healthy animals [8]. High production of ε toxin in the digestive tract often follows sudden changes in diet that disrupt the microbial balance and result in overgrowth of ε toxin-producing *C*. *perfringens* (>10^6^ CFU/g, usually 10^8^−10^9^ CFU/g). As a result, pore-forming epsilon toxin acts locally by increasing intestinal permeability and eventually it can enter the bloodstream and cause perivascular edema in tissues such as kidneys, lungs, heart and brain [1,9]. It should be noted that very few ε toxin-mediated natural diseases have been reported in humans [10–14].

With an intraperitoneal LD_50_ of ≈ 70 ng/kg in mice, ε toxin is the third most potent clostridial toxin after botulinum and tetanus toxins and is thus considered to be a potential biological weapon [3,15]. For this reason, it was classified in category B by the Centers for Disease Control and Prevention (CDC).

To date, ε toxin can be detected by several techniques, but few commercial tests are currently available, and only for veterinary diagnostic purposes. The most accepted criterion in establishing a definitive diagnosis for *C*. *perfringens* type B or D disease is the detection of major toxins, in particular ε toxin, in intestinal contents, combined with histopathological changes. Historically, clinical signs, gross *post-mortem* findings and isolation of *C*. *perfringens*, despite being useful, are considered nonspecific because they are commonly observed in non-epsilon toxin-mediated illnesses and as such cannot be used to confirm the presence of epsilon toxin [9]. Originally, the most frequently used method for the toxins detection was based upon *in vivo* toxin neutralization (i.e. mouse neutralization test [16]). To reduce costs, time and animal use, alternative approaches (molecular or immunological methods) were developed. Nowadays, most veterinary diagnostic laboratories use bacteriological techniques to isolate and identify *C*. *perfringens*, followed by detection of the toxin genes by PCR. However, such detection techniques have shortcomings: the bacteriological analysis must be performed within a few hours after death, *C*. *perfringens* can easily lose the plasmid encoding ε toxin during culture and isolation, the presence of the ε toxin gene cannot unambiguously prove the presence (and the quantity) of epsilon toxin and prototoxin and finally those techniques are time-consuming and time to results vary from several hours to several days. Thus, there have been many reports of alternative methods using immunoassays for direct ε toxin protein detection by counterimmunoelectrophoresis [16], latex agglutination test [17] and several sandwich enzyme immunoassays using either polyclonal antibodies [18–20] or monoclonal/polyclonal antibodies [16,21]. Three of these immunotechniques were compared with the mouse neutralization test [16], and results showed that there was a marked inconsistency among the four techniques to detect ε toxin in different spiked or naturally contaminated ovine intestinal contents (with presumptive or experimental enterotoxemia). The enzyme immunoassays (using either monoclonal or polyclonal antibodies for capture) were nevertheless among the techniques with the highest sensitivity (up to 0.075 mouse lethal dose per mL detected by the polyclonal immunoassay). More recently, a new technique was described using immunopurification followed by liquid chromatography monitored by mass spectrometry [22,23]. To our knowledge, there is only one company (BioX, Belgium) that commercializes kits for the detection of ε toxin. These commercial tests consist of an enzyme immunoassay that detects either ε toxin alone (used in [16,21,24]) or simultaneously alpha, beta, epsilon toxins and the bacterium *C*. *perfringens* itself, and more recently a lateral flow immunoassay that detects ε toxin alone (no scientific report using this technology to date).

However, these immunological methods are not sensitive enough to address some questions, such as the quantitative distribution of ε toxin in different tissues and fluids [21]. The objective of this study was to produce and characterize new monoclonal antibodies (mAbs) against ε toxin through affinity determination, epitope mapping and *in vitro* neutralization assays in order to develop sensitive and specific immunological tests (enzymatic or lateral flow immunoassays) directed against this toxin and new immunological tools for a better understanding of the pathological mechanisms of the disease. The immunoassays were evaluated for their suitability for the detection of ε toxin in different matrices for different purposes: intestinal content for veterinary diagnosis, milk and water for biological threat detection and serum for human diagnosis.

## Materials and methods

### Bacterial cultures

*Clostridium perfringens* strains were grown in TGY (Trypticase, 30 g/L; yeast extract, 20 g/L; glucose, 5 g/L; cysteine-HCl, 0.5 g/L, pH 7.2) in anaerobic conditions. The strains 89–87, CP-A0, CP-A4, 93R, CP48, CP47, 226–12 were isolated from lambs with enterotoxemia, 230–09 was from goats with enterotoxemia, CWC238, CWC245 were from piglets with necrotic enteritis, 180–08 and 420–13 were of human origin, 667–76 was a collection strain from the National Reference Center for Anaerobic Bacteria and Botulism, and 73–20144, 73–20115, 71–2097, NCTC3181 and NCIB10748 were collection strains. The 6-h cultures were centrifuged (8000 × *g* for 10 min) and the supernatants collected for immunoanalysis.

### Prototoxin purification and activation

The prototoxin was purified from *C*. *perfringens* type D strain NCTC2062 as previously described [25]. It was further activated by trypsin (enzyme/substrate w/w ratio of 1/100) for 30 min at room temperature (RT), leading to the fully active toxin. p-Aminobenzamidine coated on agarose (Sigma) was added to inhibit and remove protease. The purity of both prototoxin and toxin was estimated to be >90% by sodium dodecyl sulfate-polyacrylamide gel electrophoresis (SDS-PAGE). The concentration was determined using the Bio-Rad protein assay.

### Preparation of epsilon antigen

Due to the toxicity of the prototoxin (LD_50_ approximately 90 μg/kg), detoxification was required before mouse immunization. For this purpose, purified prototoxin was resuspended in phosphate-buffered saline (PBS) at 400 μg/mL, and formaldehyde was added to a final concentration of 3 mM. After incubation for 19 days at 37°C, the reaction was stopped by the addition of Tris-HCl buffer pH 8 to 100 mM final concentration, and residual formaldehyde was removed by extensive dialysis against PBS. The concentration was determined using the BCA protein assay kit (Thermo Scientific).

### Production of monoclonal antibodies

All experiments were performed in compliance with the French and European regulations on care and protection of laboratory animals (European Community [EC] Directive 86/609, Décret n° 2001–486, 6 June 2001) and with the agreements of the ethics committee of the Commissariat à l’Energie Atomique (CEtEA “Comité d’Ethique en Expérimentation Animale” n° 44) no.12-026 and 15–055 delivered to S. Simon and agreement D-91-272-106 from the Veterinary Inspection Department of Essonne (France).

Three *Biozzi* mice (bred in the animal care unit at CEA) were immunized monthly for 3 months with 20 μg of detoxified prototoxin with complete Freund’s adjuvant (foot pad injection). Mice were bled before the first immunization and two weeks after each immunization. The polyclonal anti-epsilon prototoxin response was evaluated using a specific enzyme immunoassay (see below). The mouse showing the best immune response was selected for preparation of mAbs and given a daily intravenous booster injection of 30 μg detoxified prototoxin for three days. Two days after the last boost, hybridomas were produced by fusing spleen cells with NS1 myeloma cells, according to Köhler and Milstein [26]. Hybridoma culture supernatants were screened for antibody production by enzyme immunoassay (see below). Selected hybridomas were subsequently cloned by limiting dilution. Monoclonal antibodies were produced in ascites fluid in BALB/c mice and further purified using caproic acid precipitation [27]. The purity of mAbs was then assessed by SDS-PAGE in reducing and non-reducing conditions.

### Labeling of proteins with biotin or acetylcholinesterase

Epsilon prototoxin and mAbs were labeled with biotin for use as conjugates in enzyme immunoassays. Briefly, 0.67 nmol of antibody or 6 nmol of epsilon prototoxin dissolved in 400 μL of 0.1 M borate buffer pH 9 was incubated respectively with 13.3 nmoles or 18 nmoles of biotin-N-hydroxysuccinimide ester (Sigma) dissolved in water-free DMF. After a 1-h reaction at RT, 100 μL of 1 M Tris-HCl pH 8.0 was added for 1 h at RT. Finally, 500 μL of enzyme immunoassay (EIA) buffer (see composition below) was added and this preparation was stored at –20°C until use.

The best conjugate mAbs (i.e. PεTX6 and PεTX9) were labeled with acetylcholinesterase (AChE). For this purpose, a 20-fold molar excess of N-succinimidyl S-acetylthioacetate (SATA) was added to each intact mAb for 2 h at RT with stirring in the dark. After separation by gel filtration using Sephadex G-25 fine (GE Healthcare) with 0.1 M sodium phosphate pH 6.0 containing 5 mM EDTA, the acetylated sulfhyldryl groups on the SATA-modified antibody were deprotected for 30 min at RT by the addition of 1/10 (v/v) of 1 M hydroxylamine pH 7.0, 10 mM EDTA. The final product was rapidly coupled to AChE pretreated with succinimidyl-4-(N-maleimidomethyl)cyclohexane-1-carboxylate (SMCC) as previously described [28].

### Evaluation of polyclonal response and screening of mAbs in hybridoma supernatants

Anti-epsilon prototoxin antibodies were detected in sera of immunized mice or hybridoma culture supernatants using a specific EIA. Briefly, 50 μL of serial dilutions of mouse sera or hybridoma culture supernatants in EIA buffer (100 mM potassium phosphate buffer pH 7.4, containing 0.1% bovine serum albumin, 0.15 M NaCl and 0.01% sodium azide) was transferred into microtiter plates previously coated with goat anti-mouse Ig(G+M) antibodies (Jackson Immunoresearch) together with biotinylated-epsilon prototoxin (50 μL at 100 ng/mL). After overnight reaction at 4°C, plates were washed three times (washing buffer: 10 mM potassium phosphate buffer pH 7.4, 0.05% Tween20) and 100 μL of AChE-labeled streptavidin conjugate (home-made, 2 Ellman units [EU]/mL) was added to each well. After 2-h incubation at RT followed by three washing cycles, 200 μL of Ellman’s reagent (home-made) was added and the absorbance was measured at 414 nm after 1 h [28].

### SDS-PAGE and immunoblot analysis

SDS-PAGE was performed with 12% polyacrylamide slab gels using the Laemmli protocol. Dilutions of epsilon prototoxin in 60 mM Tris-HCl pH 6.8, 2.5% β-mercaptoethanol, 2% SDS, 10% glycerol and 0.01% bromophenol blue were boiled for 10 min before migration. After electrophoresis, the antigens were transferred to Hybond-P polyvinylidene difluoride (PVDF) membrane (GE Healthcare). The PVDF sheets were processed with the SNAP-ID^™^ western blot instrument (Millipore), blocked with 0.25% milk in PBS containing 0.1% Tween 20 and probed with 4 μg/mL of each purified mAb in blocking solution for 10 min. After reaction for 10 min with peroxidase-conjugated goat anti-mouse IgG antibody in blocking solution, the blot was further stained by chemiluminescence (ECL Prime, GE Healthcare).

### Epitope mapping

Decapeptides (frameshift by one residue) were synthesized on a cellulose membrane (Intavis Bioanalytical Instruments) by the Spot method of multiple peptide synthesis using an Auto-Spot Robot ASP222 (Intavis Bioanalytical Instruments) [29,30]. In this method, all peptides are presented in the same orientation, bound to the membrane by their C-terminal residue. For the epitope mapping assays, the dried peptide membranes were treated in ethanol followed by PBS immersion and then with blocking buffer (5% milk in PBS containing 0.1% Tween 20 [PBS-Tween]). After 2 washes in PBS-Tween, the reactivity of immobilized peptides was assessed by incubation with each mAb (5 μg/mL in PBS-Tween containing 1% milk) for 30 min at RT. After 3 washes in PBS-Tween, stabilized peroxidase-conjugated goat anti-mouse IgG (Thermo Scientific) was used as secondary antibody (30 min at RT, diluted 1/2000 in PBS-Tween, 1% milk) and stained by chemiluminescence (ECL Prime, GE Healthcare).

### Determination of mAb affinity by surface plasmon resonance

The affinities of mAbs were determined by surface plasmon resonance (SPR) on a BIAcore T200 instrument (GE Healthcare, Uppsala, Sweden) by carrying out two independent multi-cycle kinetic measurements for each antibody. All analyses were performed at 25°C on a CM5 sensor chip in running buffer (10 mM HEPES, 150 mM NaCl, 3 mM EDTA, 0.05% Tween 20). Polyclonal anti-mouse IgG antibody was covalently conjugated to a CM5 sensor chip according to the manufacturer’s protocol (Mouse Antibody Capture Kit, GE Healthcare, Uppsala, Sweden). Individual mAbs (1 μg/mL) were bound to this mouse capture chip for 30 s at 5 μL/min. Each captured antibody was subjected to five consecutive threefold serial dilutions of epsilon toxin (ranging from 2.47 to 1800 nM; List Biological Laboratories) for 120 s at a constant flow rate of 30 μL/min to obtain a maximum signal of approx. 30 resonance units (RU). Dissociation was monitored over a period of 600 s before the chip was regenerated with 10 mM glycine (pH 1.7) for 180 s at a flow rate of 10 μL/min. Background binding of epsilon toxin to the reference flow cell was subtracted from signals on the active flow cell. The equilibrium dissociation constant (K_D_) was calculated using the ratio between the dissociation rate constant (k_off_) and the association rate constant (k_on_) as previously described [31] using a global Langmuir 1:1 fit (Biacore T200 Evaluation Software 3.0).

### *In vitro* neutralization assays of epsilon toxin

The *in vitro* neutralization of epsilon toxin was evaluated using the Madin Darby canine kidney (MDCK) cell viability assay. MDCK cells (from Kirsten Sandvig, Oslo) were seeded on clear flat-bottom 96-well plates (2x10^4^ cells/well) at 37°C with 5% CO_2_ in complete culture medium (DMEM supplemented with GlutaMAX, 10% fetal calf serum and 1% penicillin/streptomycin). They were allowed to adhere overnight, and then incubated with 10 nM epsilon toxin in the presence or absence of purified mAbs (ranging from 100 pM to 100 nM, diluted in PBS supplemented with 5 mM glucose and 0.1% BSA) for 3.5 h. The kinetics of pore formation were investigated by the addition of propidium iodide (5 μg/mL) in the culture medium. Fluorescence measurement was performed every 5 min and for 200 min using the FluoStar Omega (BMG Labtech) with excitation/emission at 540 nm/660 nm. Calculation of the areas under the curve of measured fluorescence over the time, in the range from 30 min to 80 min, using the FluoStar Omega “Curve Analysis” module, was used to determine the concentrations of neutralizing antibodies necessary to maintain 50% cell viability (ED_50_, effective dose at 50%).

### Intestinal contents: Sampling and preparation

Intestinal contents (from jejunum, ileum, cecum, colon and rectum) were collected from hospitalized (for non-digestive causes) and euthanized farmer’s sheep for which an autopsy was performed at the French National Veterinay School of Toulouse. These samples were kept in containers and immediately frozen (-20°C) for transport. On reception, they were aliquoted and maintained at -20°C until processed for immunoassays.

Before immunotesting, intestinal contents were either resuspended in PBS (33% W/V, for cecum, colon and rectum samples) or not (jejunum and ileum samples) and vigorously homogenized before clarification by centrifugation for 20 min at 20 000 × *g*.

### Sandwich enzyme immunoassays

#### Simultaneous immunoassay

This immunoassay was used in the first development steps, when the epsilon toxin and prototoxin were used diluted in EIA buffer.

96-well microplates (MaxiSorp^™^, Nunc) were coated overnight at RT with 200 μL of each of the different mAbs at a 10 μg/mL concentration in 0.05 M potassium phosphate buffer pH 7.4. The plates were then saturated with EIA buffer and stored at 4°C until use. Toxin samples serially diluted in EIA buffer were transferred into the washed coated microtiter plates (100 μL), together with 100 μL of conjugate anti-epsilon prototoxin mAb (either biotinylated (50 ng/mL final) for complementary binding studies or directly covalently coupled to AChE [1 EU/mL final] for final tests). After reaction at 4°C overnight (named O/N simultaneous format) or at RT for 4 h (named rapid simultaneous format), followed by washing cycles, plates containing the biotinylated conjugates were reacted for 1 h at RT with 200 μL per well of 1 EU/mL of AChE-labeled streptavidin. After 6 washes, AChE activity was detected by Ellman’s colorimetric method at 414 nm after 1 h [28].

#### Sequential immunoassay

This immunoassay format was used in a second series of experiments, when the epsilon toxin and prototoxin were titrated in complex matrices, such as culture supernatants, intestinal contents, semi-skimmed milk, tap water or human sera.

Plates were coated as described above. Clarified intestinal samples were diluted 2-fold or 6-fold in PBS containing final concentrations of 25% fetal calf serum and 0.05% Tween20. Epsilon toxin was directly spiked (or not) at different concentrations in these intestinal sample preparations or in pure milk, pure human sera, pure tap water or buffered tap water (tap water with addition of one-tenth volume of 10× EIA buffer). Culture supernatants were serially diluted in EIA buffer. Samples were transferred into the coated microtiter plates (100 μL) before overnight reaction at 4°C (for O/N sequential assays) or 2-h reaction at RT (for rapid sequential assays). After washing, the AChE-labeled antibody PεTX6 (100 μL at 2 EU/mL) was added for 2 h at RT. Immunoplates were stained as previously described. Diluted epsilon toxin standards were performed in the same buffer as for the samples, and deposited onto each immunoplate to enable quantification of epsilon toxin in samples present on the same 96-well plate.

#### Limits of detection and quantification

For all immunoassay formats, limits of detection (LoD) and quantification (LoQ) were calculated using GraphPad Prism software with a nonlinear regression model using a two-site binding saturation curve fit (total and nonspecific binding). The LoD is defined as the lowest toxin concentration giving a signal greater than the nonspecific binding (mean of eight measurements of unspiked EIA buffer/matrix) + 3 standard deviations (99.9% confidence). The LoQ is defined as the lowest toxin concentration giving a signal greater than the nonspecific binding (mean of eight measurements of unspiked EIA buffer/matrix) + 10 standard deviations (99.9% confidence).

### Immunochromatographic test

The test is based on one-step immunochromatography using mAb coupled to colloidal gold particles. Preparation of colloidal gold-labeled anti-epsilon prototoxin antibodies was performed as described previously [32]. The test strip involves i) a sample pad (Standard 14, Whatman), ii) a nitrocellulose membrane (PRIMA 40, Whatman) and iii) an adsorption pad (470 paper, Whatman), each part attached to a backing card. The detection zone involves immobilized goat anti-mouse IgG antibodies (Jackson Immunoresearch) as control line and anti-epsilon prototoxin antibodies as test line (1 mg/mL solution in 0.01 M sodium phosphate buffer pH 7.4 containing 0.15 M NaCl and 0.01% sodium azide) dispensed at 1 μL/cm using an automatic dispenser (Airjet XYZ 3050, BioDot, Irvine, USA). Saturation, drying, pad assembling and cutting of the strips were done as previously described [32]. The assay was performed at RT in a 96-well microtiter plate by mixing 100 μL/well of the toxin sample with 10 μL of 100 μg/mL colloidal gold-labeled antibody (all dilutions made in immunochromatographic [ICT] assay buffer: 0.1 M Tris-HCl pH 8 containing 0.15 M NaCl, 0.5% Tween20, 0.01% sodium azide and 1% 3-[(3-cholamidopropyl)dimethylammonio]-1-propanesulfonate [CHAPS]). After 5-min reaction of the mixture with gentle shaking, the lower part of the strip (i.e. the sample pad) was inserted into the well. The complete migration of the sample by capillarity occurred in about 15 min. A positive result appeared as two lines and a negative result as a single upper control line. The detection limit corresponded to the lowest toxin concentration showing a positive result detected by the naked eye.

For immunochromatographic assays in the different matrices, epsilon toxin and prototoxin were spiked in pure semi-skimmed milk, tap water or human plasma, all of them previously buffered, i.e. addition of one-tenth volume of 10× ICT buffer (1× final ICT buffer in all different matrices). Clarified intestinal content was previously diluted 2-fold in a final concentration of 25% fetal calf serum and 1× ICT buffer, before being spiked.

A comparison with a commercial immunochromatographic test (BIO K 176, Bio-X [former version of Bio K 388]) was performed following the instructions provided in the kit. Briefly, one spoon filled to the brim with ovine intestinal content was added and mixed with the Bio-X buffer contained in a test tube, before epsilon toxin spiking and introduction of the lower part of the strip into the prepared solution. Epsilon toxin was also directly spiked into the provided Bio-X buffer or into semi-skimmed milk or human plasma both half-diluted in Bio-X buffer, before immunochromatographic testing. The result was available in 10 min (one line as negative, two lines as positive result).

## Results

### Production and characterization of 5 monoclonal antibodies directed against epsilon toxin and prototoxin

**Fig 1:**
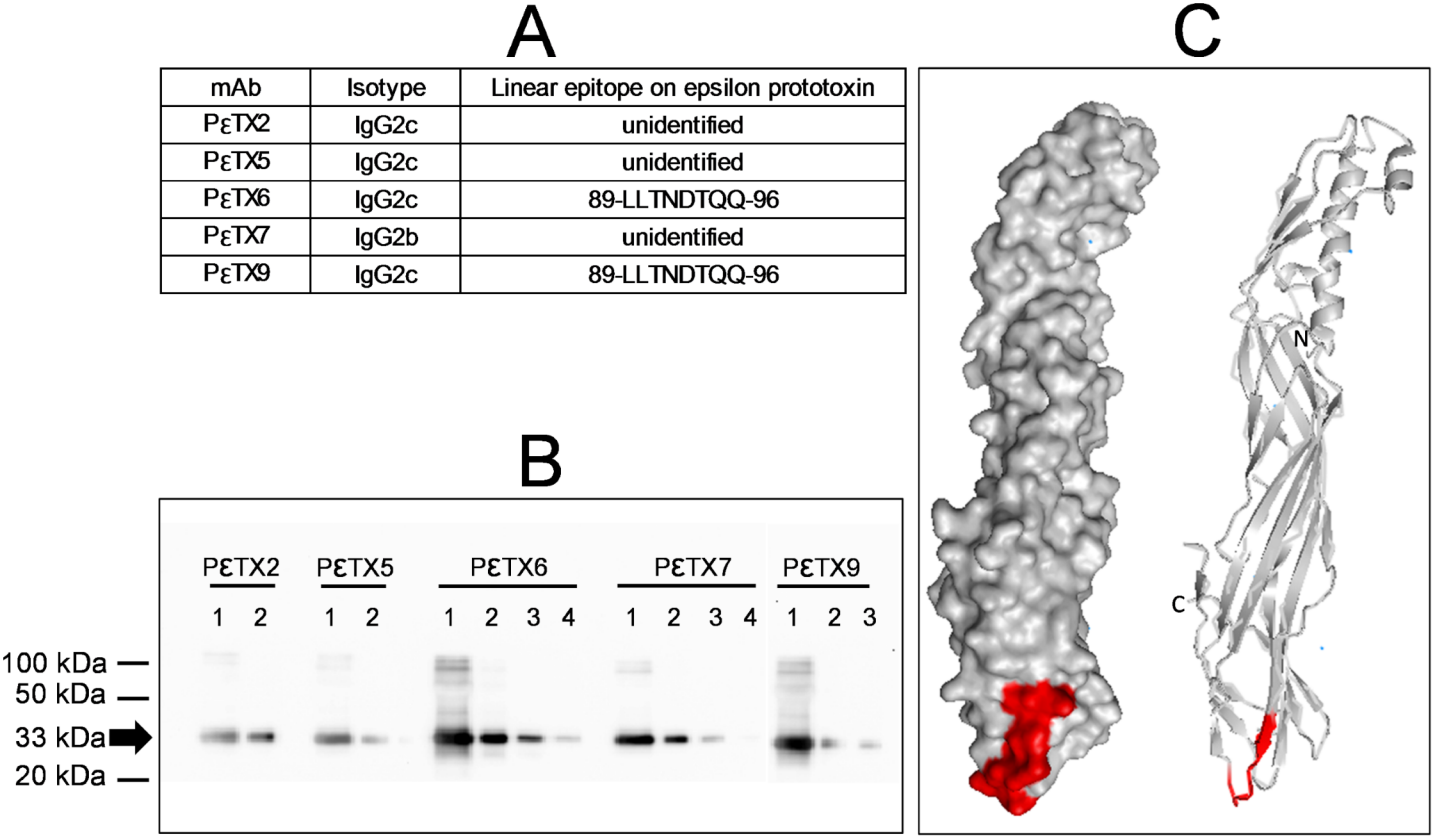
Characterization of mAbs. **A**–Linear epitopes recognized by the different mAbs were identified as described in Methods. Antibodies for which no linear epitope was identified are categorized as “unidentified”, indicating that they probably bind a conformational epitope of epsilon prototoxin. **B**–Different quantities of epsilon prototoxin (lane 1: 1 μg, lane 2: 100 ng, lane 3: 10 ng, lane 4: 1 ng) were detected by western blotting with each of the different purified mAbs produced. The arrow indicates the prototoxin band (33 kDa). **C**–Localization of the linear epitope of mAbs PεTX6 and PεTX9 on the 3D-structure of epsilon prototoxin (surface (left) and ribbon (right) diagrams of the protein with accession code 1UYJ [33]).

Antibodies were raised in *Biozzi* mice by immunization with purified detoxified epsilon prototoxin. The 5 mAbs produced were named PεTX# for “**epsilon P**roto**T**o**X**in” (Fig 1A). Purified mAbs were characterized by sandwich immunoassays (combinatorial analysis of all possible pairs of mAbs, Table 1), western blot, epitope mapping, affinity determination by SPR technology and *in vitro* neutralization assays.

**Table 1:** Colorimetric signals obtained for all combinations of mAbs used as capture and tracer antibodies in a sandwich enzyme immunoassay format.

		Capture mAb									
		PεTX2		PεTX5		PεTX6		PεTX7		PεTX9	
		ε prototoxin	ε toxin	ε prototoxin	ε toxin	ε prototoxin	ε toxin	ε prototoxin	ε toxin	ε prototoxin	ε toxin
Tracer mAb	PεTX2	**-**	**-**	**-**	**+**	**-**	**-**	**-**	**+**	**-**	**-**
	PεTX5	**-**	**-**	**-**	**-**	**+ +**	**+**	**+ +**	**+**	**-**	**-**
	PεTX6	**-**	**-**	**+ + +**	**+ + +**	**-**	**-**	**+ + +**	**+ + +**	**-**	**-**
	PεTX7	**+**	**+ +**	**+ + +**	**+ + +**	**+ + +**	**+ + +**	**-**	**-**	**+ +**	**+ +**
	PεTX9	**-**	**-**	**+ + +**	**+ + +**	**-**	**-**	**+ + +**	**+ + +**	**-**	**-**

Epsilon prototoxin or epsilon toxin (100 μL at 2.5 ng/mL in EIA dilution buffer) was simultaneously incubated overnight at 4°C with each of the 5 different biotinylated PεTX mAbs on microplates coated with each of the 5 different PεTX mAbs (see Materials and methods). Optical densities at 414 nm were measured in duplicate for epsilon toxin and protoxin (specific signal) and eight times for EIA dilution buffer (background).

The minimum detectable (MD) was calculated as the mean + 3 standard deviations from the background signal.

“**-**”is representative of a specific signal < 2 MD, i.e. noncompatible pairs of mAbs.

“**+**”, “**+ +**” and “**+ + +**” are representative of a specific signal > 2, > 5 and > 10 MD respectively, i.e. compatible and interesting pairs of mAbs.

A complementary binding study for all combinations of mAbs (Table 1) confirmed that these 5 antibodies recognize both epsilon toxin and prototoxin, in a simultaneous sandwich immunoassay. Six combinations allowed good detection of toxin at a final concentration of 2.5 ng/mL (signal > 0.5 absorbance units [AU] after 1 h), without any optimization. Monoclonal antibodies PεTX2, PεTX6 and PεTX9 are not compatible, because of their probable binding to an identical or neighboring epitope on the toxin. Two other mAbs, PεTX5 and PεTX7, bind to different epitopes, as each of them can bind simultaneously to the toxin with each of the 4 other antibodies.

The 5 mAbs also recognized epsilon prototoxin in western blot experiments (Fig 1B) with different sensitivities (1 ng for PεTX6, 10 ng for PεTX7 and PεTX9, 100 ng for PεTX2 and PεTX5). An epitope mapping (pepscan) of these 5 antibodies was performed. Two of them (PεTX6 and PεTX9) recognize the same linear epitope (89-LLTNDTQQ-96) located in the β-sandwich domain III of the protein [33] which straddles the C-terminal extremity of the final strand of the four-stranded sheet and its following loop just before the three-stranded sheet (Fig 1A and 1C). We were unable to determine the epitope of the three other mAbs, indicating that although able to bind to the partially denatured prototoxin in western blots experiments they probably recognize a conformational epitope of epsilon prototoxin. As mAb PεTX2 cannot bind ε toxin simultaneously with PεTX6 or PεTX9, PεTX2 epitope might be located near or be part of the one of PεTX6 and PεTX9.

Kinetic parameters of the five antibodies were determined by SPR biosensor technology in multi-cycle kinetics using epsilon toxin as antigen (Table 2). The dissociation constant K_D_ was calculated from the ratio of k_off_/k_on_. All antibodies exhibited similar K_D_ values in the range of 10^−9^ M, except PεTX2 and PεTX5 showing a slightly lower K_D_ of 9.6×10^−9^ and 9.8×10^−9^ M respectively. Despite similar K_D_ of the mAbs, some differences in association and dissociation rate could be observed: PεTX2, PεTX6 and PεTX9 exhibited lower dissociation rates (approx. 1×10^−4^ s^–1^) while PεTX5 and PεTX7 showed faster association (1.2×10^5^ and 2.0×10^5^ M^–1^s^–1^ respectively).

**Table 2:** Affinity constants of mAbs for epsilon toxin.

mAb	k_off_ (s^–1^)	k_on_ (M^–1^s^–1^)	K_D_ (M)
PεTX2	(1.0 ± 0.9) × 10^−4^	(1.0 ± 0.1) × 10^4^	(9.6 ± 8.2) × 10^−9^
PεTX5	(1.1 ± 0.1) × 10^−3^	(1.2 ± 0.2) × 10^5^	(9.8 ± 2.8) × 10^−9^
PεTX6	(1.2 ± 0.1) × 10^−4^	(6.2 ± 0.9) × 10^4^	(1.9 ± 0.5) × 10^−9^
PεTX7	(7.0 ± 0.9) × 10^−4^	(2.0 ± 0.5) × 10^5^	(3.7 ± 0.5) × 10^−9^
PεTX9	(1.3 ± 0.1) × 10^−4^	(6.1 ± 0.5) × 10^4^	(2.1 ± 0.4) × 10^−9^

### *In vitro* neutralization of epsilon toxin by antibodies

**Fig 2:**
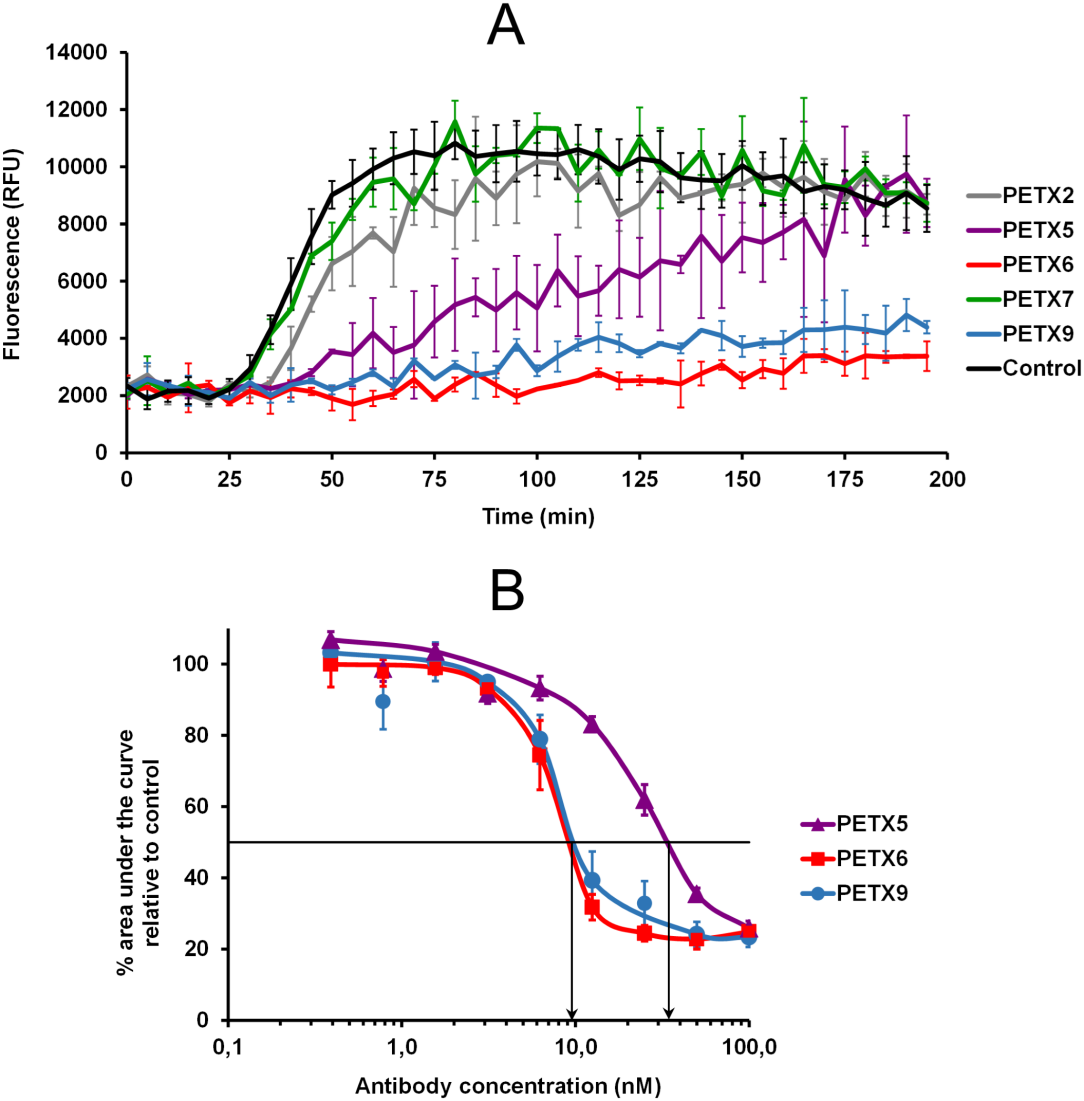
Epsilon toxin antibody neutralization effect *in vitro* using a viability assay with MDCK cells. **A**–Neutralization assay of epsilon toxin using the five mAbs. Kinetic curves of propidium iodide entry in epsilon toxin-treated MDCK cells were obtained using 10 nM epsilon toxin without mAb (control) or with 100 nM of each mAb. **B**–Comparison of inhibition of propidium iodide entry into epsilon toxin-treated MDCK cells by the three mAbs PεTX5, PεTX6 and PεTX9 using varying antibody concentrations (0.39–100 nM). Areas under the curves of measured fluorescence over time were calculated within the range from 30 min to 80 min. Error bars represent standard deviations from a duplicate for antibody neutralization assays and from ten measurements for the control (without mAb). RFU, relative fluorescence unit.

To further characterize the five mAbs produced, all antibodies were tested for their ability to neutralize epsilon toxin cytotoxicity *in vitro*. The cytotoxic dose that killed 50% of cells was determined to be around 90 nM in our conditions (i.e. 2×10^4^ cells/well and 3-h incubation with epsilon toxin). Therefore, a toxin concentration of 10 nM was used for antibody neutralization assays using 2×10^4^ cells per well. The capacity of mAbs to neutralize epsilon toxin cytotoxicity was tested using a viability assay (see Material and methods). The nuclei of MDCK cells treated with epsilon toxin alone or with non-neutralizing mAbs are readily stained with propidium iodide (PI), which is consistent with pore formation by the toxin, as opposed to viable cells treated with an antibody showing neutralizing activity towards epsilon toxin pore formation. In a preliminary PI exclusion assay using an antibody concentration of 100 nM, we found that 3 out of 5 mAbs (PεTX5, PεTX6 and PεTX9) were endowed with inhibitory activity against ETX-induced cytotoxicity (Fig 2A). Further experiments using 10 nM ETX but varying mAb concentration from 0.39 to 100 nM, confirmed their inhibitory activity. Concentrations of neutralizing antibodies necessary to maintain 50% cell viability were determined to be around 10 nM (1.5 μg/mL) for PεTX6 and PεTX9, and 30 nM (4.5 μg/mL) for PεTX5 (Fig 2B).

### Development of highly-sensitive ε toxin and prototoxin detection tests: Sandwich enzyme immunoassays and immunochromatographic tests

#### Enzyme immunoassays

**Fig 3:**
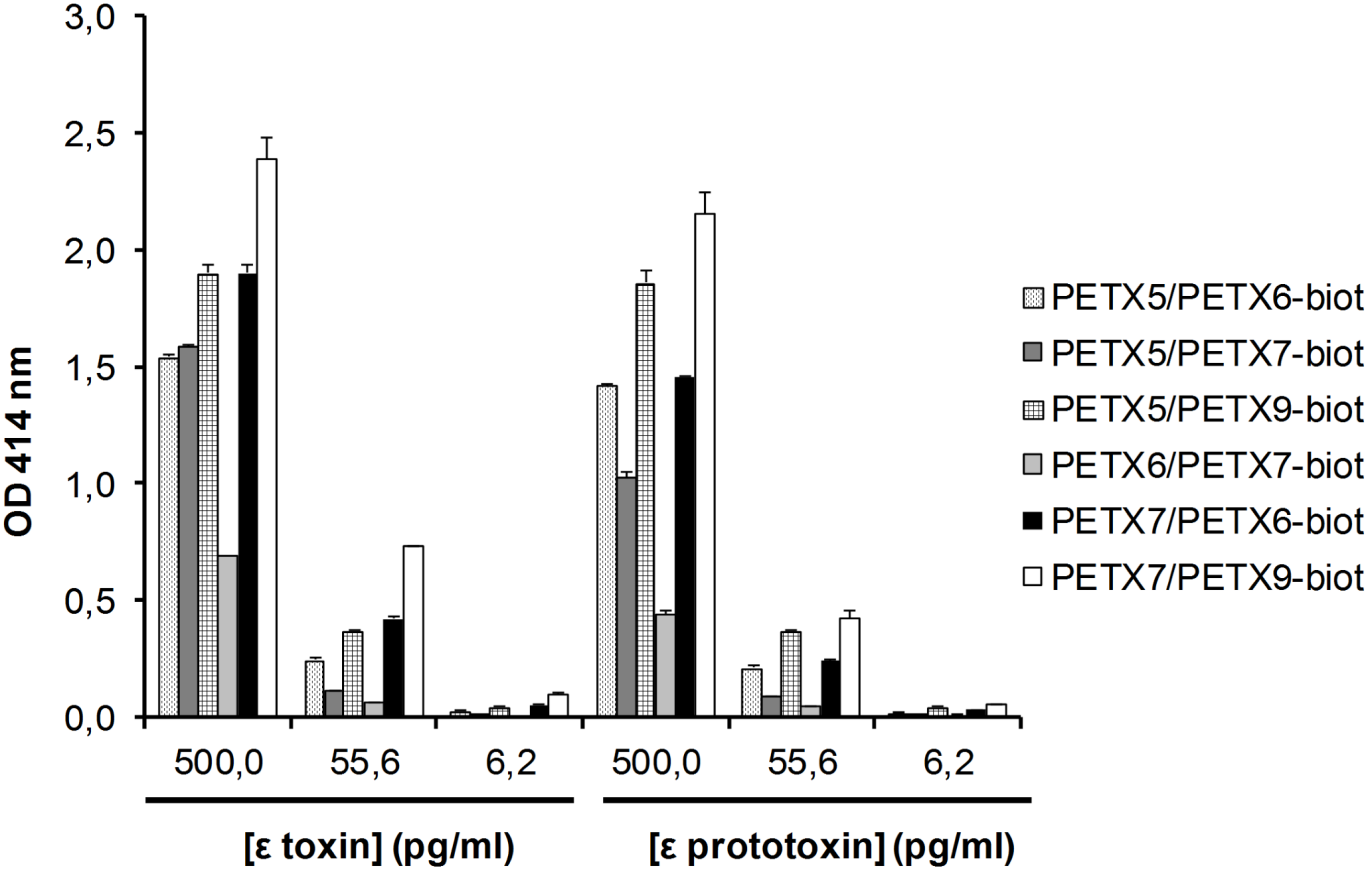
Comparison of the best selected immunoassays for the detection of epsilon toxin and prototoxin. Different concentrations of epsilon toxin and prototoxin were detected with various combinations of mAbs in an overnight simultaneous format (simultaneous incubation of toxin and biotinylated antibody on the coated immunoplate). In order to allow a direct comparison, the nonspecific binding of each pair of mAbs was subtracted. Error bars represent standard deviations from a duplicate. OD, optical density.

The 6 best sandwich enzyme immunoassays identified in the combinatorial analysis in Table 1 were further evaluated for dilution series of epsilon toxin and prototoxin (Fig 3). In a simultaneous 18-h reaction format using biotinylated antibodies as conjugate antibodies, three combinations of mAbs were selected: P*ε*TX7 as capture antibody used in combination with either P*ε*TX6 or P*ε*TX9 as biotinylated tracer, and P*ε*TX5 as capture mAb with P*ε*TX9 as conjugate antibody.

**Fig 4:**
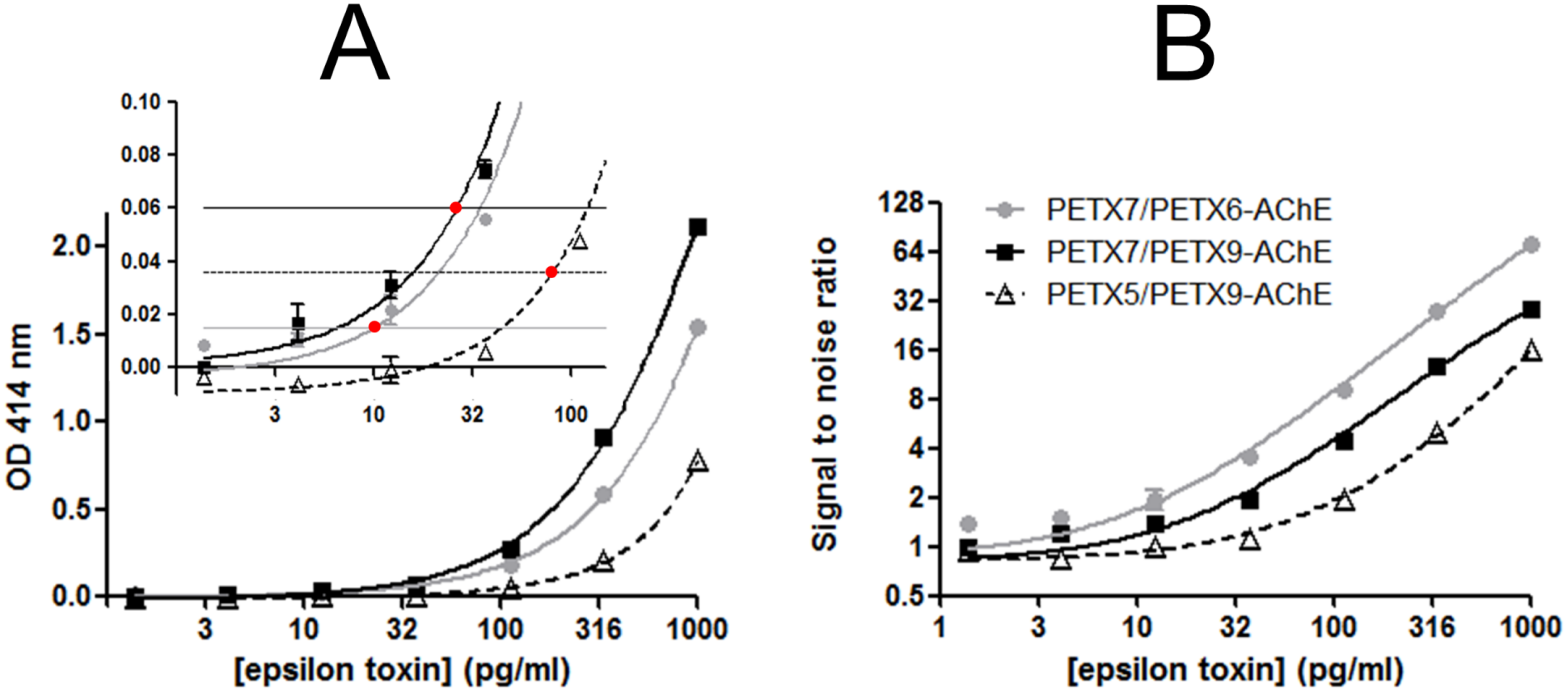
Comparison of the three best immunoassays for the detection of epsilon toxin. Different concentrations of epsilon toxin were detected with various combinations of mAbs in an overnight sequential format (where toxin was incubated alone overnight, before antibody conjugate reaction). In **A**, in order to allow a direct comparison, the nonspecific binding of each pair of mAbs was subtracted. The insert shows the low concentration part of the curve. Red points indicate the detection limit for each sandwich immunoassay. Gray, black and dotted horizontal lines represent the detection limit for the mAb combinations PεTX7/PεTX6-AChE, PεTX7/PεTX9-AChE and PεTX5/PεTX9-AchE, respectively. These detection limits were calculated as 3 standard deviations of the nonspecific binding (nonspecific binding of each pair of mAbs subtracted). In **B** is shown the signal-to-noise ratio (signal as a mean of a duplicate measurement, noise of eight measurements). Error bars represent standard deviations from the duplicate. OD, optical density.

To set up an optimized sandwich enzyme immunoassay in terms of sensitivity, specificity, signal-to-noise ratio and rapidity, the best conjugate antibodies were directly labeled with acetylcholinesterase (AChE) and the selected combinations of mAbs were evaluated with a dilution series of epsilon toxin and prototoxin in different formats (sequential versus simultaneous incubations, long versus rapid formats). The immunoassays involving P*ε*TX7 as capture antibody with AChE-conjugated P*ε*TX6 as tracer showed the best sensitivities and/or the best signal-to-noise ratio in all formats and were further selected for the enzyme immunoassay format (illustrated in Fig 4 for O/N sequential reaction format for epsilon toxin). Table 3 reports the limits of detection (LoD) and quantification (LoQ) obtained for epsilon toxin and prototoxin in EIA buffer in all formats tested. The best sensitivities were obtained for the overnight simultaneous format with performances of approximately 5 pg/mL (0.15 pM) for LoD and 15 pg/mL (0.5 pM) for LoQ. A 3-fold decrease in sensitivity was obtained for the overnight sequential format and a 6-fold decrease for both faster sequential and simultaneous formats (which reached an LoD near 30 pg/mL [0.9 pM] and a LoQ of 90 pg/mL [2.7 pM]). In all immunoassay formats tested, we could observe a slightly better sensitivity for detection of epsilon toxin in comparison with that of prototoxin.

**Table 3:** Sensitivity of the sandwich enzyme immunoassay P*ε*TX6/P*ε*TX7-AChE for epsilon toxin and prototoxin in different formats.

		Enzyme immunoassay			
		O/N simultaneous	O/N sequential	4-h simultaneous	4-h sequential
Epsilon prototoxin	LoD (pg/mL)	5.4 ± 1.8 (n = 4)	15.5 ± 3.8 (n = 4)	28.5 ± 1.9 (n = 3)	31.4 ± 6.5 (n = 3)
	LoD (pM)	0.16 ± 0.06	0.47 ± 0.11	0.86 ± 0.06	0.95 ± 0.20
	LoQ (pg/mL)	17.0 ± 4.8 (n = 4)	47.5 ± 13.5 (n = 4)	87.9 ± 7.8 (n = 3)	94.1 ± 9.0 (n = 3)
	LoQ (pM)	0.52 ± 0.14	1.44 ± 0.41	2.66 ± 0.24	2.85 ± 0.27
Epsilon toxin	LoD (pg/mL)	2.9 ± 0.9 (n = 4)	8.6 ± 0.3 (n = 3)	17.8 ± 3.0 (n = 3)	13.3 ± 3.7 (n = 6)
	LoD (pM)	0.10 ± 0.03	0.30 ± 0.01	0.62 ± 0.11	0.46 ± 0.13
	LoQ (pg/mL)	9.1 ± 2.1 (n = 4)	26.9 ± 0.9 (n = 3)	55.3 ± 11.3 (n = 3)	41.9 ± 14.9 (n = 6)
	LoQ (pM)	0.32 ± 0.07	0.94 ± 0.03	1.93 ± 0.39	1.47 ± 0.52

Four sandwich formats were evaluated: a long and a rapid simultaneous format either O/N at 4°C or 4 h at RT, and a long and a rapid sequential format (toxin was incubated first alone, either O/N at 4°C or 2 h at RT, before antibody tracer reaction for 2 h at RT). All dilutions of toxins were performed in EIA buffer.

Limits of detection (LoD, signal greater than nonspecific binding + 3 standard deviations) and quantification (LoQ, signal greater than nonspecific binding + 10 standard deviations) were calculated using GraphPad Prism software with a nonlinear regression model using a two-site binding saturation curve fit (total and nonspecific binding).

Data represent the mean of n experiments performed by the same operator using the same reagents.

AChE, acetylcholinesterase.

#### Immunochromatographic tests

**Fig 5:**
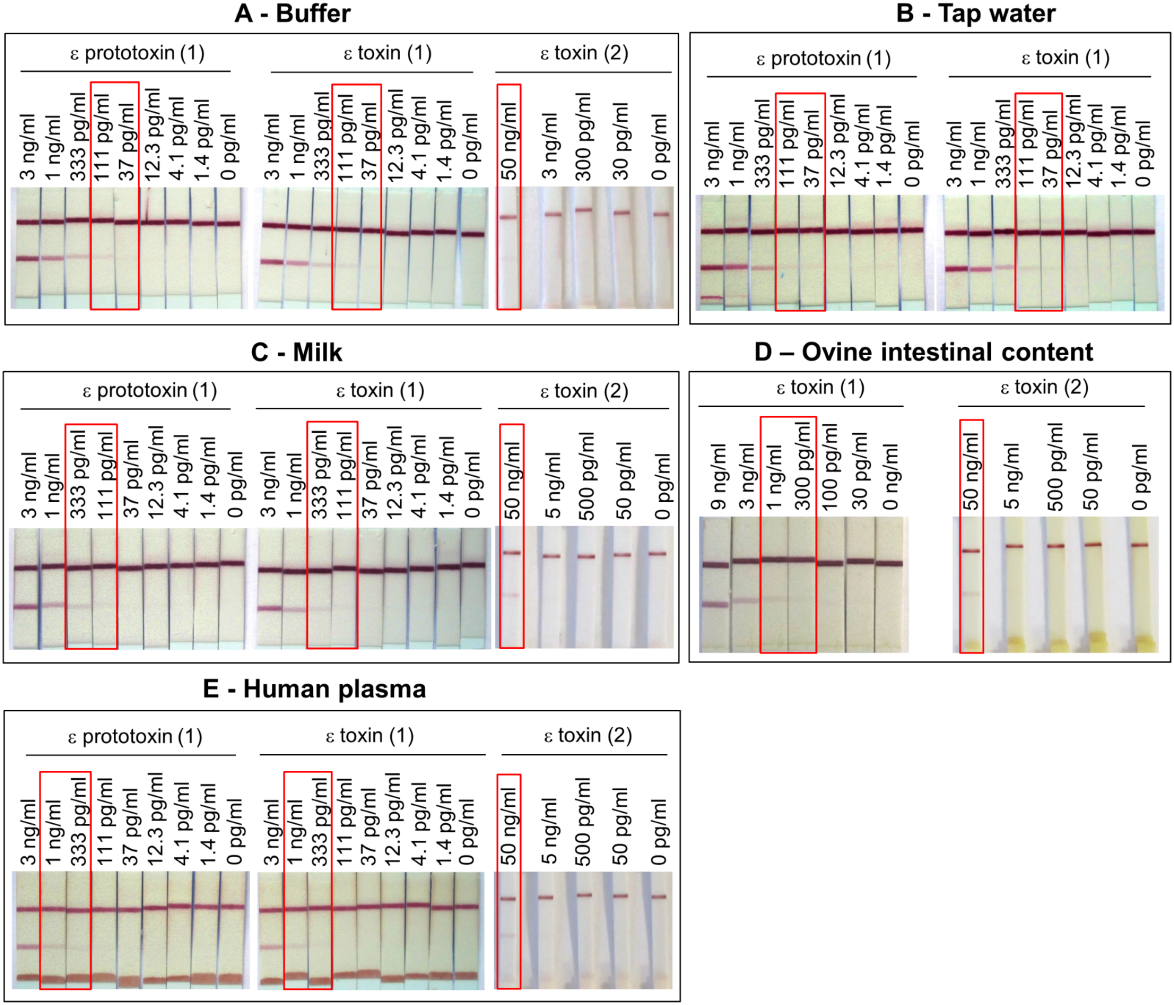
Detection of epsilon toxin and prototoxin using an immunochromatographic test. (**1**) Serial dilutions of epsilon toxin and prototoxin were prepared in immunochromatographic (ICT) buffer (**A**), buffered tap water (**B**), milk (**C**), buffered and clarified ovine jejunum content from sheep 111136 (**D**) or buffered human plasma (**E**) before detection with the lateral flow immunoassay developed using PεTX5 mAb (test line) and a goat anti-mouse immunoglobulin antibody (control line) as capture antibodies, and colloidal gold-labeled PεTX7 mAb as tracer. Milk, tap water and human plasma were previously buffered, i.e. addition of one-tenth volume of 10× ICT buffer (1× final ICT buffer in all different matrices). Clarified intestinal content was previously diluted 2-fold in a final concentration of 25% fetal calf serum and 1× ICT buffer, before being spiked. (**2**) Serial dilutions of epsilon toxin were prepared in commercial Bio-X buffer (test strips kit BIO K 176, Bio-X) (**A**), in milk previously half-diluted in commercial Bio-X buffer (**C**), in ovine jejunum content from sheep 111136 prepared following the instructions provided in the kit (**D**) or in human plasma previously half-diluted in commercial Bio-X buffer (**E**). These epsilon toxin preparations were analyzed using the commercial test strips BIO K 176 (Bio-X) following the instructions provided in the kit.

All mAb combinations were also evaluated in an immunochromatographic format for detection of epsilon toxin and prototoxin (Table 4). The best sensitivity without any background noise was obtained for the combination involving P*ε*TX5 as capture antibody with colloidal gold-labeled P*ε*TX7 conjugate. A limit of detection close to 100 pg/mL (approximately ten-fold less sensitive than immunoenzymatic test) was reached in buffer for both epsilon toxin and prototoxin in 20 min (Fig 5A). A commercial test (BIO K 176, Bio-X) has been evaluated and compared to our immunochromatographic test: it showed a limit of detection of 50 ng/mL of toxin (500-fold less sensitive) (Fig 5A).

**Table 4:** Signals obtained for all combinations of mAbs used as capture and tracer antibodies in an immunochromatographic assay format.

		Capture mAb									
		PεTX2		PεTX5		PεTX6		PεTX7		PεTX9	
		ε prototoxin	ε toxin	ε prototoxin	ε toxin	ε prototoxin	ε toxin	ε prototoxin	ε toxin	ε prototoxin	ε toxin
Tracer mAb	PεTX2	**-**	**-**	**-**	**+**	**-**	**-**	**+**	**+ +**	**-**	**-**
	PεTX5	**-**	**+**	**-**	**-**	**+ +**	**+ +**	**+ +**	**+ +**	**+ +**	**+ +**
	PεTX6	**+**	**+**	**+**	**+ +**	**+ +**	**+**	**+ +**	**+ +**	**+**	**+**
	PεTX7	**-**	**+**	**+ +**	**+ +**	**+ +**	**+ +**	**-**	**-**	**+ +**	**+ +**
	PεTX9	**+**	**+**	**+**	**+**	**+**	**+**	**+ +**	**+ +**	**+**	**+**

Immunochromatographic (ICT) buffer (100 μL), epsilon prototoxin or epsilon toxin (100 μL at 5 ng/mL in ICT buffer) was reacted for 5 min with each of the 5 different colloidal gold-labeled PεTX mAbs (10 μL of a 100 μg/mL solution in ICT buffer). Each mixture was then migrated on strips coated with each of the 5 different PεTX mAbs (see Materials and methods).

“**-**”is representative of a negative test line and a positive control line in the presence of epsilon toxin/prototoxin, i.e. noncompatible pairs of mAbs.

“**+**” and “**+ +**” are representative of a positive or highly positive test line, respectively, associated with a positive control line, in the presence of epsilon toxin/prototoxin.

Gray squares are representative of nonspecific signals, i.e. a positive test line and a positive control line for the ICT buffer migration assay in the absence of epsilon toxin/prototoxin.

In conclusion, the sensitivities of the rapid and long immunoenzymatic tests and the immunochromatographic test are excellent in buffer. Their robustness and performances were further evaluated in matrices that could be encountered in diagnostic procedures as well as biothreat events (culture supernatants, food matrices (milk and water), plasma and ovine intestinal contents).

### Specific detection of ε toxin in culture supernatants

To evaluate the specificity of the two developed immunotests (the enzyme immunoassay and the lateral flow immunoassay), 18 culture supernatants from different *Clostridium perfringens* strains from the French National Reference Center for Anaerobic Bacteria and Botulism were tested in both formats in a blind study. These supernatants did (or did not) contain epsilon toxin, together with many other toxins.

Results obtained with both the O/N sequential enzyme immunoassay and the immunochromatographic test were similar (Table 5) in terms of positive/negative identification and quantification of epsilon toxin in supernatants. When comparing these results with the decoding (right column), one mismatch was revealed for the NCTC3181 strain, supposed to be an epsilon toxin-secreting B toxinotype. However, after PCR control, it appears that the NCTC3181 *Clostridium perfringens* strain had lost the toxinogenic plasmid encoding epsilon toxin (but not the other major toxins), explaining the apparent discrepancy. This blind study clearly demonstrated the very good specificity of the two immunotests, with no false-positives or -negatives, and no cross-reactivity for the other toxins produced by *Clostridium perfringens*.

**Table 5:** Specificity evaluation of the two immunotests: Blind testing of supernatants from different *Clostridium perfringens* toxinotypes.

Strain #	Enzyme immunoassay		Immunochromatography		*Clostridium perfringens* toxinotype
	Result	Measured toxin concentration	Result	Measured toxin concentration	
**89–87**	**NEGATIVE**	**< LoD**	**NEGATIVE**	**< LoD**	A (alpha)
**73–20144**	**NEGATIVE**	**< LoD**	**NEGATIVE**	**< LoD**	C (alpha, beta)
**CWC238**	**NEGATIVE**	**< LoD**	**NEGATIVE**	**< LoD**	C (alpha, beta)
**NCTC3181**	**NEGATIVE**	**< LoD**	**NEGATIVE**	**< LoD**	B (alpha, beta, **epsilon**, delta) …with lost epsilon toxin-encoding plasmid
**CP-AO**	**POSITIVE**	**119 μg/mL**	**POSITIVE**	**≈ 100 μg/mL**	D (alpha, **epsilon**)
**180–08**	**NEGATIVE**	**< LoD**	**NEGATIVE**	**< LoD**	A (alpha)
**71–2097**	**POSITIVE**	**459 ng/mL**	**POSITIVE**	**≈ 300 ng/mL**	B ((alpha, beta, **epsilon**)
**CP-A4**	**POSITIVE**	**3.4 μg/mL**	**POSITIVE**	**≈ 3 μg/mL**	D (alpha, **epsilon**)
**93R**	**NEGATIVE**	**< LoD**	**NEGATIVE**	**< LoD**	A (alpha, beta2)
**CWC245**	**NEGATIVE**	**< LoD**	**NEGATIVE**	**< LoD**	C (alpha, beta)
**CP48**	**POSITIVE**	**1.7 μg/mL**	**POSITIVE**	**≈ 3 μg/mL**	D (alpha, **epsilon**)
**CP47**	**NEGATIVE**	**< LoD**	**NEGATIVE**	**< LoD**	A (alpha)
**73–20115**	**POSITIVE**	**235 ng/mL**	**POSITIVE**	**≈ 300 ng/mL**	B (alpha, beta, **epsilon**)
**NCIB10748**	**NEGATIVE**	**< LoD**	**NEGATIVE**	**< LoD**	E (alpha, iota)
**667–76**	**NEGATIVE**	**< LoD**	**NEGATIVE**	**< LoD**	alpha negative
**230–09**	**POSITIVE**	**2.4 μg/mL**	**POSITIVE**	**≈ 3 μg/mL**	D (alpha, **epsilon**)
**226–12**	**POSITIVE**	**4.6 μg/mL**	**POSITIVE**	**≈ 3 μg/mL**	B (alpha, beta, **epsilon**)
**420–13**	**NEGATIVE**	**< LoD**	**NEGATIVE**	**< LoD**	A (alpha)

18 culture supernatants from different *Clostridium perfringens* strains from the French National Reference Center for Anaerobic Bacteria and Botulism (M. Popoff) were tested and titrated, in a blind study, using the O/N sequential enzyme immunoassay and the immunochromatographic test. The decoding is shown in the right column.

LoD, limit of detection.

### Detection of ε toxin and prototoxin in intestinal contents for veterinary diagnosis

To validate the use of the two developed immunoassays with field veterinary samples, artificially contaminated (spiked) intestinal ovine contents from healthy sheep were tested. Previously, adjustments for intestinal sample preparation, sample dilution and sandwich enzyme immunoassay format were required. As the intestinal content matrix is not homogeneous but made of dietary fragments in a variable quantity of bile salts, clarification is needed. Different techniques were evaluated and compared: combined use of tissue homogenizer with glass or ceramic beads, filtration with 0.8 μm or 0.45 μm filter units and/or centrifugation. The best results were obtained for jejunum and ileum contents with direct clarification by centrifugation for 20 min at 20 000 × *g*, while 0.75 g from cecum, colon or rectum contents were previously resuspended in 2.25 mL of PBS before the same clarification process (data not shown). Different dilution buffers were then evaluated and compared before enzyme immunotesting in order to reduce the solid phase-bound IgG desorbing activity well documented for stool [34,35]. Dilutions in PBS containing 25% fetal calf serum and 0.05% Tween20 reduced the matrix effect (data not shown). The most sensitive sandwich immunoassay format for the detection of epsilon toxin or prototoxin spiked in such complex intestinal matrices was the sequential one (data not shown). This sequential format (toxin sample incubated alone overnight, before antibody conjugate reaction) limited the matrix effect, partly due to inhibition of AChE catalytic activity in some complex matrices.

Twenty-one ovine intestinal contents from 13 healthy sheep (13 jejunum, 2 ileum, 2 cecum, 2 colon and 2 rectum contents) were clarified and spiked (or not) with epsilon toxin before immunotesting with the O/N sandwich sequential immunoassay PεTX7/PεTX6-AChE. Table 6 illustrates the heterogeneity in performance in detection of epsilon toxin spiked in ovine intestinal contents, and the importance of performing a minimum dilution of the intestinal content to reduce the impact of the matrix effect. When the intestinal content was only half diluted before immunotesting, the matrix effect considerably affected the sensitivity of the test (LoD and LoQ) for more than half of the samples (12/21), whereas this effect was reduced to 4/21 samples when the intestinal contents were diluted 6-fold. It is clear that intestinal contents interfere to different degrees with the sandwich immunoassay by inhibiting the signals and/or by increasing the nonspecific binding. When the intestinal contents are diluted 6-fold before immunotesting (the lowest dilution for which the inhibition is abolished), the limit of detection for spiked epsilon toxin is at least 50 pg/mL, i.e. 300 pg/mL in the original intestinal contents.

**Table 6:** Limits of detection and quantification of epsilon toxin spiked in different ovine intestinal contents.

	Sequential O/N PeTX7/PeTX6-AChE			
	1/2 diluted intestinal sample		1/6 diluted intestinal sample	
Sample	LoD (pg/mL)	LoQ (pg/mL)	LoD (pg/mL)	LoQ (pg/mL)
Jejunum T325	25.5	73.1	9.0	24.2
Jejunum T203	45.8	140.6	26.2	80.6
Jejunum 111136	14.2	40.5	4.8	11.9
Jejunum S216	11.9	30.4	6.5	17.0
Jejunum S218	21.7	66.6	6.1	17.8
Jejunum S254	26.7	79.8	7.0	20.9
Jejunum S263	6.6	21.8	6.0	34.8
Jejunum T180	49.1	136.3	21.2	62.9
Jejunum T336	16.5	54.8	6.2	19.7
Jejunum T346	10.6	30.8	7.5	23.4
Jejunum T349	98.7	282.0	18.0	53.2
Jejunum 130010	610.0	N.C.	4.8	13.6
Ileum 130010	162.6	383.8	6.3	17.1
Cecum 130010	2.4	6.9	5.9	17.6
Colon 130010	5.3	14.9	4.1	11.5
Rectum 130010	3.3	7.7	3.9	10.5
Jejunum 130033	18.1	62.1	5.9	23.1
Ileum 130033	29.8	93.1	12.6	39.3
Cecum 130033	3.0	5.8	3.1	6.8
Colon 130033	3.0	8.2	2.6	7.1
Rectum 130033	2.8	7.2	2.7	5.4
Dilution buffer	7.4 ± 3.6 (n = 11)	21.8 ± 11.2 (n = 11)		

Serial dilutions of epsilon toxin were performed in different matrices: either in dilution buffer (PBS containing 25% fetal calf serum and 0.05% Tween20) or in different clarified ovine intestinal contents diluted 2-fold or 6-fold in dilution buffer. All these dilutions were measured with the overnight sequential enzyme immunoassay PεTX7/PεTX6-AChE (see Materials and methods).

Theoretical limits of detection (LoD = mean of nonspecific binding + 3 standard deviations) and quantification (LoQ = mean of nonspecific binding + 10 standard deviations) were calculated using GraphPad Prism software with a nonlinear regression model using a two-site binding saturation curve fit (total and nonspecific binding).

N.C., not calculable.

The lateral flow immunoassay was also evaluated for the detection of spiked epsilon toxin in 2-fold diluted clarified ovine intestinal contents. The limit of detection was between 300 pg/mL and 1 ng/mL (Fig 5D) (i.e. up to 2 ng/mL in the original intestinal contents), while the sensitivity was at least 25 fold inferior (50 ng/mL) for a commercial test strip kit with the same spiked samples.

### Detection of ε toxin and prototoxin in food and plasma for biosafety purposes

Milk, tap water and human plasma were chosen as representative matrices for biological threat detection and diagnosis. The effect of these matrices on the detection of spiked epsilon toxin and prototoxin was evaluated using both the fast and long sequential enzyme immunoassays PεTX7/PεTX6-AChE as well as the lateral flow immunoassay PεTX5/colloidal gold-labeled PεTX7.

**Fig 6:**
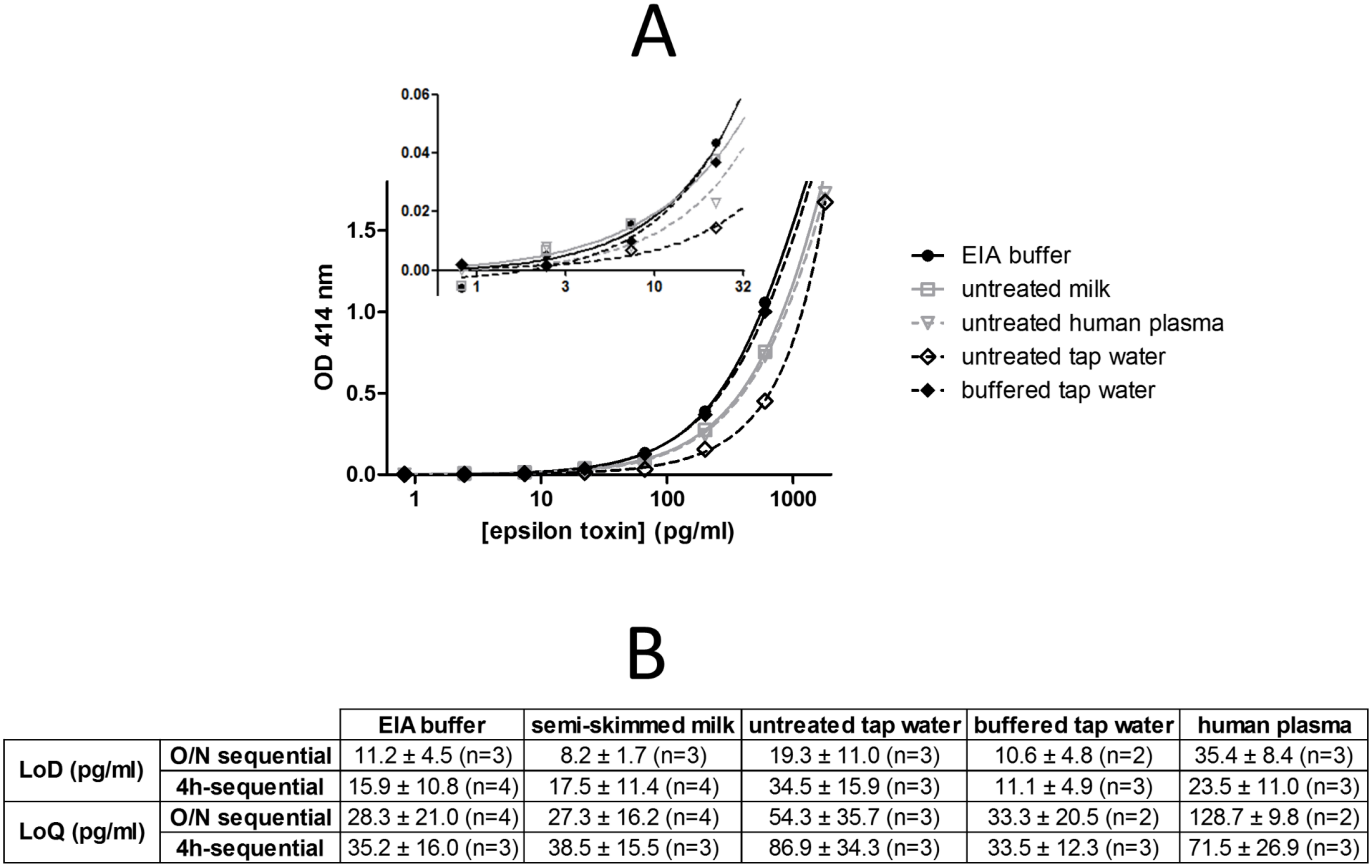
Detection of epsilon toxin spiked in EIA buffer, milk, tap water and human plasma using sequential enzyme immunoassay PεTX7/PεTX6-AChE. Serial dilutions of epsilon toxin were performed in different matrices: EIA buffer, pure semi-skimmed milk, pure human plasma, untreated pure tap water and buffered tap water. These dilutions were detected using the overnight (**A** and **B**) or the 4-h (**B**) sequential enzyme immunoassay PεTX7/PεTX6-AChE (see Materials and methods). In **A**, in order to allow a direct comparison, the nonspecific binding was subtracted. The inserts show the low-concentration part of the curve. Error bars represent standard deviations for a duplicate measurement. Theoretical limits of detection (LoD = mean of nonspecific binding + 3 standard deviations) and quantification (LoQ = mean of nonspecific binding + 10 standard deviations) were calculated using GraphPad Prism software with a nonlinear regression model using a two-site binding saturation curve fit (total and nonspecific binding).

As shown in Fig 6 for the sequential immunoassays, the sensitivities of epsilon toxin detection were similar in EIA buffer, milk and buffered tap water. A slight decrease in sensitivity was observed when toxin was diluted in pure human plasma and untreated tap water (4-fold decrease at the maximum). Nevertheless, the matrix effect with untreated tap water can be avoided by adding EIA buffer to a 1× final concentration either with the fast or with the long sequential immunoassay (Fig 6B). Similar results were obtained with the epsilon prototoxin (data not shown).

Using the lateral flow immunoassay, few differences were observed between buffer, buffered tap water and pure buffered milk, with sensitivities reaching approximatively 100 pg/mL (Fig 5A, 5B and 5C). Some constituents of human plasma were clustered at the bottom of the dipstick, probably retaining epsilon toxin, and thus producing a 10-fold decrease (1 ng/mL) in sensitivity in this undiluted matrix (Fig 5E). In comparison, a 50 ng/mL sensitivity was obtained in previously 2-fold diluted milk and human plasma using the commercial Bio-X test strip kit.

## Discussion

Epsilon toxin from *Clostridium perfringens* is the third most potent clostridial toxin and is considered as a potential biological weapon, classified in category B by the CDC. In the case of a bioterrorist attack, a rapid, sensitive and specific detection method will be required to monitor food/water or human contamination by this toxin. There is plethora of scientific reports that address the development of detection tests and counter-measures against diverse potential bioterrorism agents. However, very few specifically document the development of detection tests for epsilon toxin and prototoxin in food or water and diagnostic methods for human body fluids [22,23]. This study describes the development of highly sensitive rapid tests able to address this question.

Five mAbs directed against common epitopes of ε toxin and prototoxin were produced and characterized. Thanks to their high affinity, they allowed the development of highly sensitive and specific detection tests for epsilon toxin and prototoxin for use in laboratories (96-well enzyme immunoassay performed in at least 4 h) as well as in the field (lateral flow immunoassay performed in 20 min). Two formats of enzyme immunoassays, a fast version (4 h) and a longer version (overnight incubation) were developed and reached, respectively, theoretical detection limits of at least 30 pg/mL (0.9 pM) and 15 pg/mL (0.5 pM) and quantification limits of at least 90 pg/mL (2.7 pM) and 45 pg/mL (1.4 pM) for both epsilon toxin and prototoxin in buffer. In tap water and milk, these sensitivities are unaffected. However, as expected in more complex matrices, the sensitivities decrease from less than a 4-fold diminution in human plasma up to 30-fold for ovine intestinal contents. Despite this, the sensitivity remains excellent, even in the shorter 4-h format.

The sensitivity of the lateral flow immunoassay reaches 100 pg/mL for ε toxin and prototoxin in buffer, in 20 min. This detection limit is unaffected in tap water, slightly reduced in milk (approximately 300 pg/mL) and reduced to 1 ng/mL in human plasma and 2 ng/mL in original ovine intestinal contents (with a 2-fold dilution required). The capture and conjugate antibodies selected for this test format differ from those of the enzymatic test: comparison of the association and dissociation rate constants of antibodies shows only slight differences and the selection of the best antibody pairs is more related to the lowest nonspecific binding they are generating rather than the specific signal, for which they are almost equivalent.

These limits of detection are at least 5-fold better than those described in the literature. To our knowledge, the most sensitive sandwich ELISAs described to date to detect epsilon toxin involve polyclonal antibodies as capture and/or tracer antibodies [16,18,19]. Nagahama et al. reported a minimum detectable near 0.1 ng/mL for purified ε toxin in buffer with a home-made 3.5-h polyclonal sandwich ELISA [19]. However, even if this ELISA was evaluated with standard *C*. *perfringens* supernatants and *C*. *perfringens* strains from patients or foods, the sensitivity of this test was not evaluated in more complex matrices. Another sandwich ELISA, performed in 5.5 h, also involving two polyclonal antibodies as capture (sheep) and primary antibody (rabbit), reached a limit of detection of 2 ng/mL for ε toxin in buffer, and 4 ng/mL for the toxin spiked in ovine intestinal content [18]. Finally, two other sandwich ELISAs were compared with two other techniques (counterimmunoelectrophoresis and mouse neutralization test) [16]. The two sandwich enzyme immunoassays were found to be the most sensitive techniques for detection of epsilon toxin in different artificially spiked ovine body fluids, with an advantage in sensitivity for the home-made polyclonal/polyclonal 18-h sandwich ELISA (with reported LoD of 32 ng/mL [20] and 0.075 MLD_50_/mL [16]) over the commercial Bio-X monoclonal/polyclonal test performed in 2.5 h with a sensitivity of 25 MLD_50_/mL [16].

One study [8] reported the presence of sub-toxic concentrations of epsilon toxin in some part of the intestinal contents of 46 of 100 healthy sheep. In the present work, 21 samples of intestinal contents from 13 healthy sheep were analyzed and, although some of them gave high original signals, we were unable to link these high signals with the demonstrative presence of epsilon toxin. Indeed, attempts to correlate these intrinsic elevated signals with the presence of endogenous epsilon toxin or prototoxin at subtoxic concentrations in these healthy sheep were unfruitful as PCR experiments with extracted DNA from these matrices were negative regarding the presence of toxinogenic *Clostridium perfringens* (data not shown). Further experiments and improvements are required to address this question better.

The high affinity of our mAbs has previously proven helpful for the selective immuno-enrichment of epsilon toxin in different artificially spiked matrices, which is an essential step prior to mass quantification by liquid chromatography monitored by mass spectrometry (LC-MS/MS) [23]. Thanks to this efficient sample immuno-preparation and the specificity of our antibodies, a limit of quantification close to 1 ng/mL was reached for ε toxin spiked in different matrices (buffer, milk, human urine and plasma) in the presence of other toxins (ricin and staphylococcal enterotoxin B) in multiplex high-resolution targeted mass spectrometry [22].

Moreover, three out of five of the mAbs produced showed an *in vitro* neutralizing activity towards epsilon toxin pore formation on MDCK cells. Two of these neutralizing antibodies (PεTX6 and PεTX9) recognize the same linear epitope (89-LLTNDTQQ-96) located in the β-sandwich domain III of the protein [33] at the C-terminal extremity of the four-stranded sheet and its following loop just before the final three-stranded sheet. To our knowledge, this epitope was never previously identified as important for cytotoxicity [36]. Concentrations of our neutralizing antibodies necessary to maintain 50% cell viability were determined to be in the 1 to 10 μg/mL range, i.e. in the same range as in previous studies reporting antibodies that neutralize the cytotoxic activity of epsilon toxin [37–39]. However, this neutralization ability of our mAbs remains to be confirmed *in vivo*, together with their mechanisms of action. These three newly described neutralizing mAbs are new tools that might help for a better understanding of the cytotoxic mechanisms of the epsilon toxin and open the way to the development of medical countermeasures needed to inhibit the activity of the toxin.

Since we did not have access to clinical samples, the tests performances have been evaluated using spiked samples. Even if the results cannot be extrapolated directly to the ones we might obtain with true clinical samples, it must be underlined that our tests present a sensitivity never described previously in the literature, and at least 25-fold experimentally better than the only commercially available lateral flow immunoassay, with such complex matrices as intestinal contents. To sum up, our enzyme immunoassays are the first monoclonal/monoclonal Ab sandwich ELISAs described to date for the detection of ε toxin and prototoxin, with sensitivities at least 5-fold better in buffer and up to 100-fold better (in intestinal content) than the best ones described. This is also the first time that a lateral flow immunoassay for ε toxin and prototoxin field detection has been described with this level of sensitivity, which is at least 25-fold better than a commercial test strip (Bio-X) and in only 20 min. The lateral flow immunoassay is well suited for initial, very fast (less than 30 min) detection of the toxin in field samples and ELISA assays can be used as confirmation tests in the case of positive or doubtful results with lateral flow immunoassay.

To conclude, the highly sensitive immunotests developed in this study might be useful in addressing some investigations, such as epidemiological studies to determine the threshold for ε toxin toxicity in intestinal contents or other body fluids and its quantitative distribution in different tissues and fluids, thereby shedding light on the disease.

## Abbreviations

AChE: acetylcholinesterase
EIA: enzyme immunoassay
mAb: monoclonal antibody
